# Integrative Analysis of the Genomic and Immune Microenvironment Characteristics Associated With Clear Cell Renal Cell Carcinoma Progression: Implications for Prognosis and Immunotherapy

**DOI:** 10.3389/fimmu.2022.830220

**Published:** 2022-05-23

**Authors:** Enyu Lin, Ping Zhu, Chujin Ye, ManLi Huang, Xuechao Liu, Kaiwen Tian, Yanlin Tang, Jiayi Zeng, Shouyu Cheng, Jiumin Liu, Yanjun Liu, Yuming Yu

**Affiliations:** ^1^Department of Urology, Guangdong Provincial People’s Hospital, Guangdong Academy of Medical Sciences, Guangzhou, China; ^2^Shantou University Medical College, Shantou, China; ^3^Department of Immunology, School of Basic Medical Science, Southern Medical University, Guangzhou, China; ^4^Department of Operating Room, Cancer Hospital of Shantou University Medical College, Shantou, China; ^5^Department of Gastrointestinal Surgery, Affiliated Hospital of Qingdao University, Qingdao, China; ^6^The Second School of Clinical Medicine, Southern Medical University, Guangzhou, China

**Keywords:** clear cell renal cell carcinoma (ccRCC), genomic alteration, tumor immune microenvironment, immunotherapy, multi-omics analysis

## Abstract

Unlike early clear cell renal cell carcinoma (ccRCC), locally advanced and metastatic ccRCC present poor treatment outcomes and prognosis. As immune checkpoint inhibitors have achieved favorable results in the adjuvant treatment of metastatic ccRCC, we aimed to investigate the immunogenomic landscape during ccRCC progression and its potential impact on immunotherapy and prognosis. Using multi-omics and immunotherapy ccRCC datasets, an integrated analysis was performed to identify genomic alterations, immune microenvironment features, and related biological processes during ccRCC progression and evaluate their relevance to immunotherapy response and prognosis. We found that aggressive and metastatic ccRCC had higher proportions of genomic alterations, including *SETD2* mutations, Del(14q), Del(9p), and higher immunosuppressive cellular and molecular infiltration levels. Of these, the Del(14q) might mediate immune escape in ccRCC *via* the VEGFA-VEGFR2 signaling pathway. Furthermore, immune-related pathways associated with ccRCC progression did not affect the immunotherapeutic response to ccRCC. Conversely, cell cycle pathways not only affected ccRCC progression and prognosis, but also were related to ccRCC immunotherapeutic response resistance. Overall, we described the immunogenomic characteristics of ccRCC progression and their correlations with immunotherapeutic response and prognosis, providing new insights into their prediction and the development of novel therapeutic strategies.

## Introduction

Clear cell renal cell carcinoma (ccRCC) is a common urological malignancy and a leading cause of cancer-related deaths worldwide (1). ccRCC TNM stage classification is based on tumor progression and directly affects treatment modality and prognosis (2, 3). Localized ccRCC can be treated by nephron-sparing surgery, whereas advanced ccRCC is difficult to treat and presents with a poor prognosis and high recurrence rate. The recent development of immune checkpoint inhibitors (ICIs) markedly improved the prognosis of patients with metastatic ccRCC, but only a few of them experienced significant and lasting benefits (4). Therefore, identifying the biological features associated with ccRCC progression and response to immunotherapeutic agents could improve patient assessment, treatment selection, and prognosis.

ccRCC has relatively unique genomic characteristics, including Del(3p), Amp(5q), and somatic mutations in *VHL*, *PBRM1*, *SETD2*, and *BAP1* (5). Although Del(3p) and *VHL* mutation are hallmark features of ccRCC, these genomic alterations showed no clear association with clinical outcomes. In contrast, Del(9p) and Del(14q) were identified as potent risk factors of metastasis and mortality in ccRCC (6). Furthermore, several pan-cancer analyses showed that ccRCC had significant inflammatory features and was one of the tumor types with the highest T cell infiltration (7, 8). However, unlike other tumors, high CD8^+^ tumor-infiltrating lymphocytes (TILs) were not associated with a good prognosis in ccRCC (9).

Recent studies demonstrated breakthrough results with ICIs for advanced ccRCC and became the first-line recommended regimen when combined with targeted therapy (2, 3). PD-L1 expression has been used to predict response to ICIs in other tumors like lung cancer (10). However, increasing evidence suggests that ccRCC response to immunotherapy was independent of the extent of CD8^+^ T cell infiltration and PD-L1 expression (11) and could not be predicted by tumor mutational burden (TMB), as done in some other cancers (12, 13). The unique genomic features and immune microenvironmental characteristics of ccRCC have complex implications for treatment response and prognosis. The relationships between them should be disentangled.

This study aimed to describe the immunogenomic landscape during ccRCC progression using multi-omics data on ccRCC from The Cancer Genome Atlas (TCGA) and International Cancer Genome Consortium (ICGC) cohorts and immunotherapeutic response in an immunotherapy cohort. The study results could help understand the potential mechanisms behind the disease progression and resistance to immunotherapy and act as a reference when searching for more effective prognostic and immunotherapeutic response predictors and optimizing treatment strategies for ccRCC.

## Methods

### Patient Cohorts and Data Preprocessing

Data on somatic mutations, somatic copy number alterations (SCNAs), gene expression, and clinical information of patients with ccRCC were obtained from the TCGA database (https://portal.gdc.cancer.gov/repository) and named the TCGA cohort. Data from the ICGC portal (https://dcc.icgc.org/projects/RECA-EU) included somatic mutations, gene expression, and clinical information of patients with ccRCC, and named the ICGC cohort. After excluding samples with unspecified American Joint Committee on Cancer (AJCC) staging, which were used in subsequent studies, the TCGA and ICGC cohorts included, respectively, 348 and 72 patients with multi-omics data (**Table S1**). The patients were assigned one of the following phenotypes based on their TNM staging to more precisely characterize the aggressive and metastatic traits of the ccRCC: localized ccRCC (Stages I and II; T_1-2_N_0_M_0_), aggressive ccRCC (Stage III; T_1-2_N_1_M_0_ or T_3_N_any_M_0_), and metastatic ccRCC (Stage IV; T_4_N_any_M_0_ or T_any_N_any_M_1_). Raw reads in the two cohorts were transformed into transcripts per million (TPM) to allow for more direct comparability of gene expression between samples. Besides, normalized gene expression data and published clinical information on patients with metastatic ccRCC were obtained from the anti-PD-1 therapy clinical trial cohorts Checkmate 009 and Checkmate 025 (12 and 75 patients, respectively) (11). Patients with objective response or stable disease with tumor shrinkage and progression-free survival (PFS) of at least six months or with disease progression and a PFS of less than three months were grouped as having clinical benefit (CB) or no clinical benefit (NCB), respectively, as described by Braun et al. (11). Clinical information of patients with kidney renal papillary cell carcinoma (KIRP) and chromophobe renal cell carcinoma (KICH) was obtained from the TCGA database. Ethics approval and informed consent were not applicable, given that the data used in this study were publicly available.

### Prognostic Correlation With Tumor Phenotypes

Kaplan-Meier analysis was used to estimate the prognostic impact of the AJCC staging and phenotypes on the overall survivals (OS) and recurrence-free survivals (RFS). Univariate and multivariate analyses by the Cox proportional hazard (CoxPH) model assessed whether AJCC staging and phenotypes were independent predictors of poor OS and RFS in patients with ccRCC and computed hazard ratios for each phenotype relative to the localized phenotype in the CoxPH models. We used the time-dependent receiver operating characteristic (ROC) area under the curve (AUC) to plot the differences between the analyzed CoxPH models to assess the predictive accuracy over time.

### Comparison of Mutations Among Phenotypes

After excluding silent mutations, the TCGA and ICGC cohorts’ mutation files were encoded as a binary gene-sample matrix, with 1 indicating the presence of a non-silent mutation in a specific gene in a specific sample. Mutations with frequencies higher than 10% were defined as recurrent mutations and the remainder as low-frequency mutations. Mutations with a frequency greater than 2% were compared based on the gene’s mutational status to assess mutated genes associated with tumor progression. Two-sided Fisher’s exact test compared the mutation frequencies in the various phenotypes. Significantly different mutations were compared between every two phenotypes by the Mann-Whitney *U* test.

### Comparison of SCNAs Among Phenotypes

Individual copy number estimates, segmented per-sample arm-level, focal-level, and gene-level copy ratios, were identified using GISTIC 2.0 (14). The SCNAs were classified as amplification (≥2), gain (=1), loss (–1), and deletion (–2) according to the discrete values generated by GISTIC. Subsequently, the association of SCNAs with phenotype was tested as described above for mutation analysis. Negative copy-number values were set to zero for amplification-centered analysis, while positive copy-number values were set to zero for deletion-centered analysis to define amplification and deletion events explicitly.

### Analysis of the Association Between SCNAs and Target Gene Transcription

Analysis of the transcriptional regulatory relationship between SCNAs and the target genes included basal expression levels and correlation analysis between the target genes and SCNAs and between gene expression and tumor progression. Expression was considered strongly affected by SCNAs if it was significantly correlated with the SCNAs in all phenotypes; weakly affected if it was correlated only with some phenotypes; otherwise it was considered not applicable. The correlation between gene expression and tumor progression (i.e., phenotypes) was defined as strong if significant in both cohorts, weak if significant in only one cohort, or else considered not applicable. The basal target gene expression levels were measured by correlating between the tumor and normal tissues. Target genes were labeled as having high or low basal expression based on the significant change direction from the normal tissue expression and whether the changing trend was consistent in both cohorts; otherwise, they were considered moderate basal expression levels.

### Quantification of Microenvironment Cell Abundance and Immunomodulators

CIBERSORT, MCPCounter, quantTIseq, and ImmuCellAI were used to quantify tumor-infiltrating immune cells in ccRCC samples from the TCGA and ICGC cohorts based on TPM-normalized gene expression data (15–18). Phenotypes differed in a type of immune cell if the same trend was observed in the results of at least half of the tools. The CIBERSORT results were processed following the protocol of Thorsson et al. (19) to compare with the other tools’ results. We drew on published immunomodulator lists and integrated them to characterize tumor immune escape (19–22). The newly integrated immunomodulators were categorized as immunoinhibitory factors, immunostimulatory factors, chemokines and receptors, interleukins and receptors, interferons and receptors, and antigen-presenting molecules (**Table S2**). The immunomodulators’ corresponding copy number levels came from the gene-level SCNAs generated by GISTIC. Cytolytic activity and *IFNG* and *GZMB* transcript levels were used to measure the anti-tumor immune activity (23, 24), where the cytolytic activity scores were calculated as the geometric mean of *GZMA* and *PRF1* gene expressions (25).

### Calculation of the Immunogenicity Indicators

TMB, fraction of genome altered (FGA), neoantigen load, intra-tumoral heterogeneity (ITH) score, cancer testis antigen (CTA) score, and homologous recombination deficiency (HRD) score were used to measure the tumor immunogenicity (22, 26–30). Neoantigen load was defined as the number of peptides that single-nucleotide variant and insertion/deletion (indel) predicted to bind to major histocompatibility complex (MHC) proteins and induce anti-tumor adaptive immunity. HRD score was the sum of three independent indicators’ scores reflecting genomic scar formation. The neoantigen load and HRD score data were retrieved from the **Supplemental Materials** in the study by Thorsson et al. (19). TMB was calculated as the sum of all non-synonymous mutations per megabase. FGA was defined as the proportion of genomes with |log_2_ copy-number >0.2| relative to the genome size. CTAs are genes whose expression is typically restricted to the human germline but aberrantly expressed in malignant tumors and could activate anti-tumor immune responses (31). CTA scores per sample were obtained from single-sample gene set enrichment analysis (ssGSEA) performed for a set of 201 cancer-testis genes curated by the cancer-testis database using the GSVA package with default settings (31, 32). ITH scores were defined as subclonal genomic fractions generated by ABSOLUTE (19). ABSOLUTE was run using default parameters on segmented copy-number data and mutation data.

### Differential Expression and Functional Enrichment Analysis

Raw count and normalized expression data were imported, respectively, to DESeq2 and limma R package with default settings for differential gene expression analysis among phenotypes in the TCGA and ICGC cohorts and between groups with CB and NCB in the immunotherapy cohort, wherein genes with *P* < 0.05 were considered differentially expressed (33, 34). Gene set enrichment analysis (GSEA) was conducted using clusterProfiler with default parameters. Differentially expressed genes were ranked by −log_10_ (*P* value) × sign(log_2_ (fold change)) as input for the GSEA (35). The enrichment analysis used pathway terms of Hallmark and Wikipathway gene signatures from MSigDB (version 7.4, https://www.gsea-msigdb.org/gsea/msigdb/). Pathways with *P* < 0.05 were considered enriched. The enrichment direction was determined by the normalized enrichment score sign. ssGSEA was applied to evaluate the pathway enrichment scores of the samples. The intersection of the leading-edge gene of the enriched pathway between phenotypes where this pathway differed significantly was taken, and their union between the TCGA and ICGC cohorts was taken, which was taken as the thinned gene set to recalculate the ssGSEA scores (**Table S9**).

### Statistical Analysis and Data Visualization

Analysis of variance or Wilcoxon rank-sum test was used to compare the distribution of continuous variables between groups. For multiple variable analysis between groups, all the variables were normalized to 1−10 range, and odds ratio was calculated by binary logistic regression. Categorical variables were analyzed using Fisher’s exact test. Mann-Whitney *U* test and LSD test were used to assess multiple comparisons. Associations between variables were assessed by the Spearman’s rank, Pearson, or Point-biserial correlation test, as appropriate. ssGSEA scores were normalized to between 0 and 1 before comparison among groups. ssGSEA scores of each pathway were classified into high-level and low-level groups according to the median value. Kaplan-Meier survival analysis with the log-rank test was performed using the R package survival. Cox proportional hazard regression was used to identify independent prognostic factors by the R package glmnet. The factors’ relative prognostic values were compared by receiver operating characteristic (ROC) analysis using the R package timeROC (36), and the AUC was used to assess predictive power. Statistical significance was set at *P* < 0.05. Data visualization was done by the R packages ggplot2 and circlize (37). All statistical analyses were performed with R statistical software (v4.1.2, R Core Team, R Foundation for Statistical Computing, Vienna, Austria).

## Results

### Aggressiveness and Metastasis are Independent Risk Factors for Mortality and Recurrence of ccRCC

**Figure 1:**
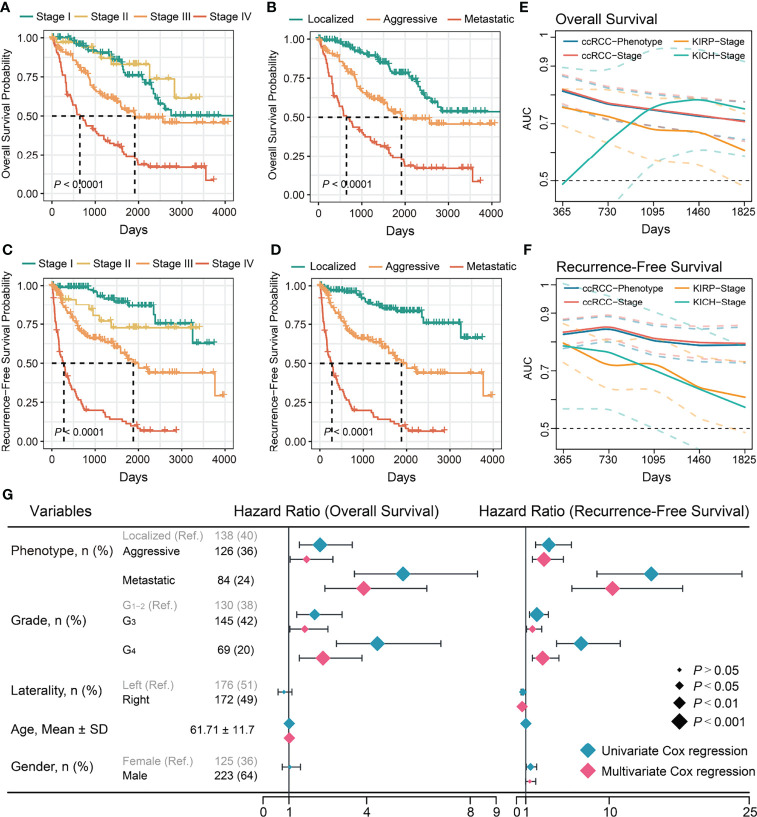
Evaluation of prognostic value of stages and phenotypes of ccRCC in TCGA cohort. **(A)** Kaplan–Meier curves of OS for stages of ccRCC. **(B)** Kaplan–Meier curves of OS for phenotypes of ccRCC. **(C)** Kaplan–Meier curves of RFS for stages of ccRCC. **(D)** Kaplan–Meier curves of RFS for phenotypes of ccRCC. **(E)** Time-dependent area under the ROC curve of stages and phenotypes for the OS in ccRCC and others. **(F)** Time-dependent area under the ROC curve of stages and phenotypes for the RFS in TCGA cohort and others. Ref: Reference group. **(G)** Forest plot of the univariate and multivariate Cox regression analysis of phenotypes and other clinical information for OS and RFS.

Survival analysis results showed that AJCC staging could predict the patient OS and ccRCC recurrence ***(*** **Figures 1A, C*****;*** **S1A*****)***, but could not distinguish between Stage I and Stage II patients in OS and RFS (P_OS_ = 0.487; P_RFS_ = 0.127; **Table S3*****)***. OS and RFS were significantly different between the ccRCC phenotypes ***(*** **Figures 1B, D*****;*** **S1B*****)***.

Univariate regression analysis showed that the aggressive and metastatic phenotypes had a significantly higher risk of mortality and recurrence than the localized phenotype ***(*** **Figure 1G*****).*** Multivariate regression analysis confirmed they were independent risk factors for mortality and recurrence of ccRCC ***(*** **Figure 1G*****)***. Subsequently, the ccRCC phenotype predictive efficacy of OS and recurrence was assessed. Time-dependent ROC analysis showed that the ccRCC phenotype had good predictive power of OS (AUC_TCGA_ = 0.71–0.81; AUC_ICGC_ = 0.66–0.81); and recurrence (AUC_TCGA_ = 0.79–0.85) within 5 years, similar to those of the AJCC stages ***(*** **Figures 1E**, **S1C**; **Table S4*****)***. We further evaluated the TNM staging predictive ability for KICH and KIRP prognoses as they used the same set of staging criteria as ccRCC. Interestingly, the AJCC stage could not distinguish well between good and poor OS or recurrence of KICH and KIRP ***(*** **Figures S1D–G**, **Table S3*****)***, and its predictive power for OS (AUC _KICH_ = 0.49–0.79; AUC _KIRP_ = 0.61–0.76) or recurrence (AUC _KICH_ = 0.61–0.80; AUC _KIRP_ = 0.57–0.79) was poor and not stable within 5 years ***(*** **Figures 1E, F*****)***. Overall, ccRCC showed more significant survival and recurrence differences between tumor progression phenotypes than other common kidney cancers. Aggressive and metastatic characteristics influence the choice of treatment modality, prognosis, and recurrence of patients with ccRCC. Therefore, further work is necessary to investigate the relevant factors affecting ccRCC progression.

### Distinct Genomic Alteration Landscapes Drive ccRCC Progression

**Figure 2:**
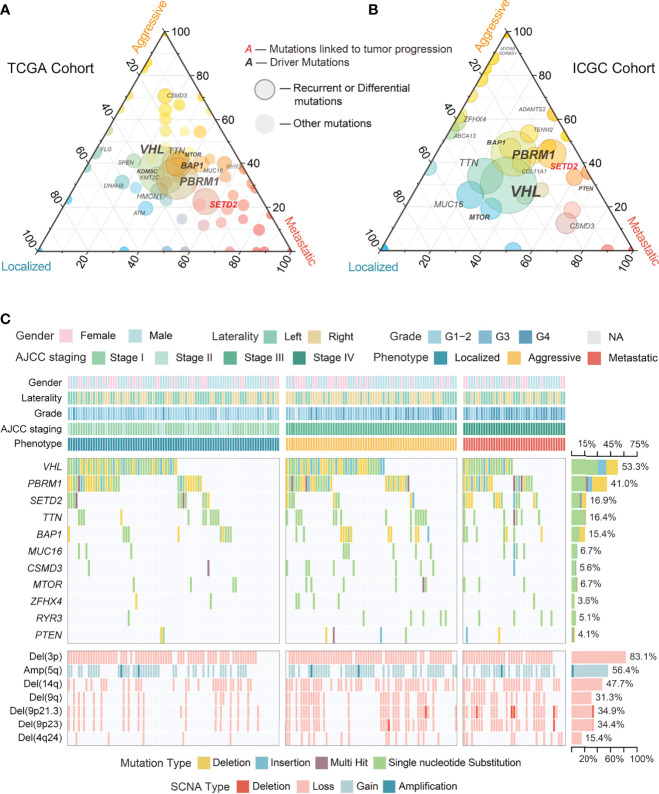
Overview of genomic alterations among phenotypes in ccRCC. **(A)** Ternary plot showing proportions of mutations among phenotypes of ccRCC in TCGA cohort. **(B)** Ternary plot showing proportions of mutations among phenotypes of ccRCC in ICGC cohort. **(C)** Waterfall plot with recurrent and differential mutations and SCNAs among phenotypes of ccRCC in TCGA cohort.

Somatic genomic alterations, including mutations and SCNAs, are important tumor initiation and progression drivers (38, 39). Common recurrent mutations (>10%) in the TCGA and ICGC cohorts integrated data included *VHL*, *PBRM1*, *SETD2*, *TTN*, and *BAP1*, of which only *SETD2* differed significantly among phenotypes (**Figures 2A, B**; **S2A**; **Tables S5–6**). *RYR3* and *PTEN* differed significantly in low-frequency mutations (<10%) but showed inconsistency in the two cohorts. We evaluated 12 driver somatic mutations in ccRCC based on the findings of Bailey et al. (40). The results showed that only *SETD2* mutations were associated with ccRCC progression (**Figures 2A, B**; **S2A**), implying that most driver mutations may be closely associated with tumorigenesis rather than ccRCC progression. However, survival analysis showed that *SETD2* mutations were not related to prognosis, while *BAP1* mutations were related to poor prognosis (**Figure S2B**). This finding could be explained by the large differences in the potential mechanisms through which they affect tumor progression and prognosis. Further multiple comparison tests found that *SETD2* mutations discriminated between localized and non-localized ccRCC, but not between aggressive and metastatic ccRCC (**Tables S5–6**). Interestingly, the above analysis did not identify somatic mutations present only in localized ccRCC.

**Figure 3:**
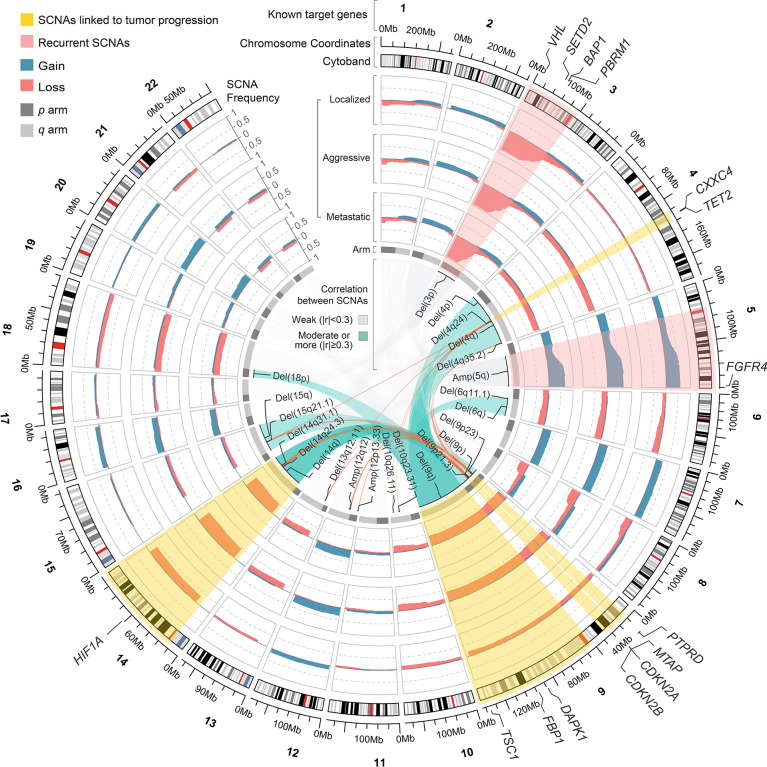
Circular plot showing the recurrent and differential SCNAs and their correlation among phenotypes of ccRCC in TCGA cohort.

In contrast to somatic mutations, SCNAs often affect a series of genes. In the TCGA cohort, Del(3p) and Amp(5q) were the most common recurrent SCNAs in ccRCC (**Figures 2C**, **3**), with similar proportions among phenotypes and no association with prognosis (**Figures 3**, **S2B**; **Table S7**), implying that they may simply be drivers of tumor initiation. High-frequency arm-level SCNAs [Del(14q) (47.7%) and Del(9q) (31.3%)] and focal-level SCNAs [Del(9p21.3) (34.9%), Del(9p23) (34.4%), and Del(4q24) (15.4%)] were more common in aggressive and metastatic ccRCC (**Figure 3**) and associated with poor prognosis (**Figure S2B**). Similar to somatic mutations, these SCNAs could not effectively discriminate between aggressive and metastatic ccRCC (**Table S7**), suggesting that they might drive ccRCC progression but not distant metastasis. Furthermore, significantly occurring SCNAs were found in all phenotypes except localized ccRCC (**Figures 2C**, **3**). Further analysis found a moderate positive correlation between the deletions on chromosome 9 and those on chromosomes 4 and 14 (**Figure 3**; **Table S8**).

**Figure 4:**
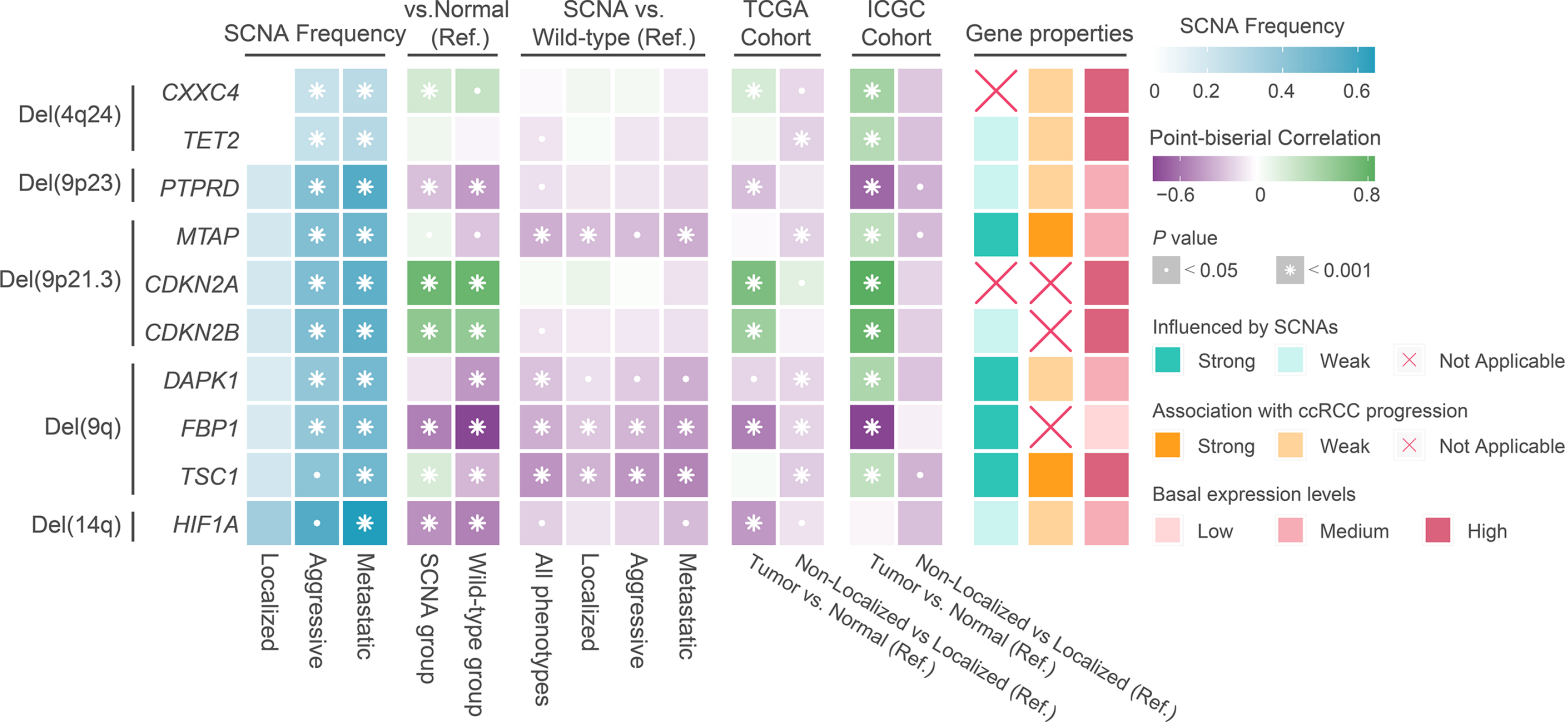
The result of transcriptional regulatory relationship between SCNAs and target genes. Ref: Reference group.

To understand the potential mechanisms by which SCNAs drive ccRCC progression, we analyzed the correlation between transcript levels of well-known genes with SCNAs and SCNA levels and ccRCC progression. We found that the transcript levels of *HIF1A*, a target of Del(14q) in ccRCC, were similar to those in normal tissues and weakly influenced by SCNAs or phenotype (**Figure 4**). The transcript levels of *DAPK1*, *FBP1*, and *TSC1*, well-known targets on Del(9q), were significantly influenced by Del(9q). Of these, only *TSC1* was strongly associated with tumor progression (**Figure 4**). Furthermore, lower mean expression levels of *FBP1* were observed in ccRCC in both cohorts than in normal tissues, while the opposite was true for *TSC1*. *CDKN2A* and *CDKN2B*, well-known tumor suppressors at 9p21.3, had higher transcript levels in ccRCC than normal tissue. These were not affected by SCNAs and tumor progression (**Figure 4**). The expression of *MTAP*, another tumor suppressor at 9p21.3, was strongly associated with SCNAs and tumor progression, although its mean level was similar to that in the normal tissue (**Figure 4**). Collectively, *MTAP* appears to be a more suitable target for Del(9p21.3) in ccRCC. The expression of *PTPRD* at 9p23 was lower in ccRCC than in normal tissue, and weakly correlated with SCNAs and tumor progression. *CXXC4* and *TET2* at 4q24 were equally potential targets for tumor suppression (41, 42). Strangely, their transcript levels in ccRCC were weakly influenced by SCNAs and not associated with tumor progression (**Figure 4**). Moreover, their mean expression levels were significantly higher in ccRCC than in normal tissues, implying that they might act as tumor suppressors in ccRCC, but that their expression was particularly influenced by regulatory factors other than SCNAs.

### Dynamic Changes in the Patterns of Immune Escape During ccRCC Progression

Schreiber et al. presented the concept of tumor immune editing and described two possible pathways involved in tumor immune escape. One was the extrinsic immune escape, i.e., exhaustion of effector TILs, high infiltration of immunosuppressive cells, and high levels of immunosuppressive cytokines and immune checkpoint molecules; the other was the intrinsic immune escape, i.e., loss of tumor antigen expression and tumor antigen-presenting capacity (43). Based on this theoretical framework, we first compared the extrinsic immune escape pattern between the ccRCC phenotypes.

**Figure 5:**
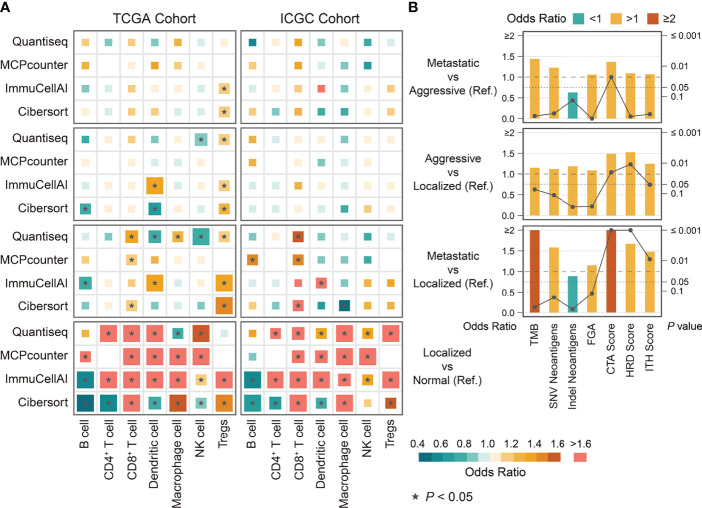
The differences in immune cells and immunogenicity indicators among phenotypes of ccRCC derived from binary logistic regression. **(A)** The differences in immune cells among phenotypes of ccRCC in TCGA and ICGC cohort. **(B)** The differences in immunogenicity indicators among phenotypes of ccRCC in TCGA cohort. Ref: Reference group.

**Figure 6:**
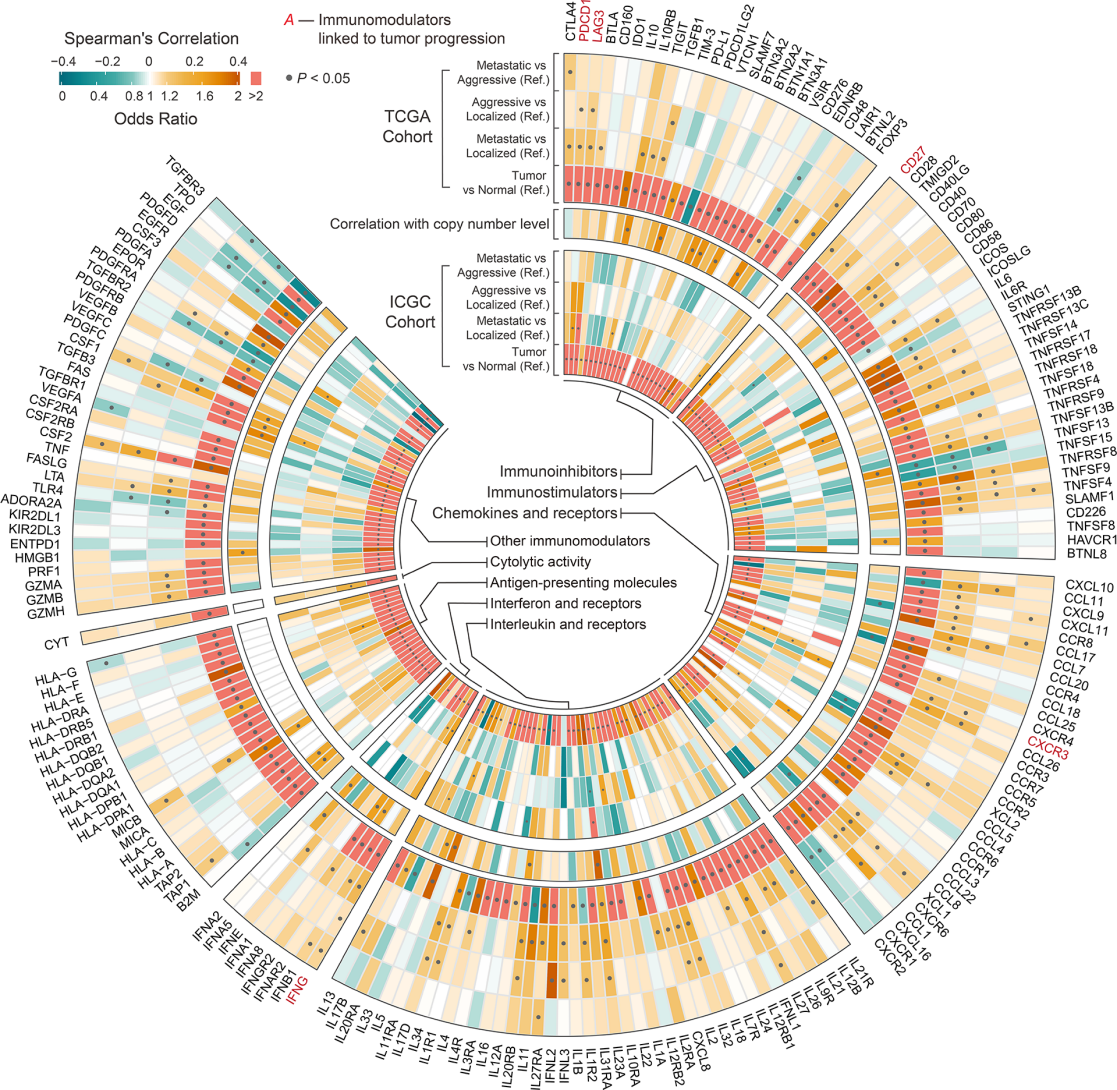
Circular plot showing the differences in immunomodulators among phenotypes and between tumor and normal tissue of ccRCC. Outer circle indicates the differences in immunomodulators among phenotypes and between tumor and normal tissue of ccRCC in TCGA cohort derived from univariable binary logistic regression. Middle circle indicates the correlation between the expression of immunomodulators and the corresponding SCNAs derived from Spearman’s analysis. Inner circle indicates the differences in immunomodulators among phenotypes and between tumor and normal tissue of ccRCC in ICGC cohort derived from univariable binary logistic regression. Ref: Reference group.

We compared the estimated levels of tumor-infiltrating immune cells in the various ccRCC phenotypes using four immune infiltration quantification tools. CD8^+^ TILs and Tregs showed higher abundance in aggressive and metastatic ccRCC in both cohorts. Levels of B, natural killer (NK), and CD4^+^ cells did not change significantly during tumor progression (**Figure 5A**). However, the estimated levels of macrophages and dendritic cells (DCs) differed too much between cohorts and quantification tools to be assessed. We compared the immune cell levels between localized ccRCC and normal tissue to assess the degree of immune cell infiltration in early ccRCC. The results showed that early ccRCC already showed higher levels of CD8^+^ TILs, CD4^+^ TILs, Tregs, NK cells, DCs, and macrophages (**Figure 5A**). Furthermore, aggressive and metastatic ccRCC had high expression of immunosuppressive molecules, including PDCD1 and LAG-3, which indirectly reflected exhaustion of TILs (**Figure 6**). Classical immunosuppressive cytokines, including vascular endothelial growth factor (VEGF), transforming growth factor-β (TGF-β), indoleamine 2,3-dioxygenase (IDO), and interleukin (IL)-10, and indicators of effector T-cell killing activity, including GZMB and cytolytic activity, did not change during ccRCC progression (**Figure 6**). However, aggressive and metastatic ccRCC had high expression levels of CD27, IFNG, and CXCR3 (**Figure 6**), which were implicated in antitumor immune activation (44, 45).

We next investigated the potential mechanisms of intrinsic immune escape in ccRCC progression. The results showed that CTA score, HRD score, and ITH score in aggressive and metastatic ccRCC were significantly higher than in the other phenotypes, whereas all phenotypes had similar levels of TMB, FGA, and neoantigen (**Figure 5B**). Of these, only CTA score significantly differed between aggressive and metastatic ccRCC. Corresponding survival analysis showed that ccRCC patients with high CTA scores, HRD scores and ITH scores presented shorter recurrence-free and overall survival time (**Figure S3**). Moreover, all MHC-related antigen-presenting molecules were similar among the ccRCC phenotypes, a result SCNAs could not explain (**Figure 6**). We evaluated the correlation between indicators of immunogenicity and extrinsic immune escape to examine whether the high immunogenicity of aggressive and metastatic ccRCC was a contributing factor to immune cell infiltration. Surprisingly, the CTA score, ITH score, and HRD score showed no obvious correlation with immune cell infiltration, antitumor immune activity, or expression of immune checkpoint molecules (**Figure S4A**).

### Biological Processes Associated With the Aggressiveness and Metastasis of ccRCC

**Figure 7:**
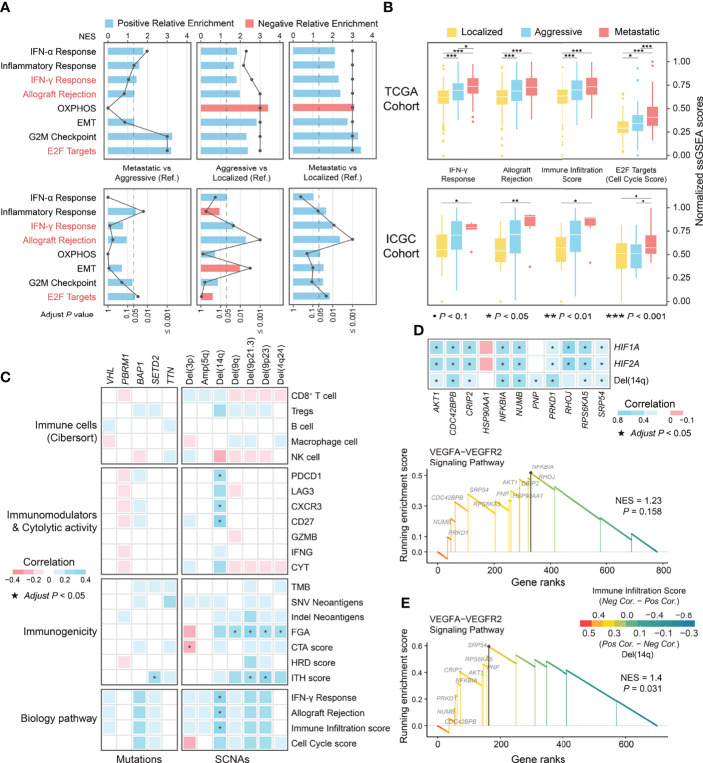
The differential biological processes among phenotypes in ccRCC and correlation of genomic alterations and immune-related indicators. **(A)** The differences in biological processes among phenotypes of ccRCC derived from GSEA. **(B)** Boxplot showing the differences in normalized ssGSEA scores of differential biological processes among phenotypes of ccRCC. **(C)** Correlation of genomic alterations and indicators related to immune escape and biological processes. **(D)** GSEA plot of the enrichment pathway on genes at 14q associated with immune infiltration score and correlation between leading edge genes with indicators including SCNA levels and expression of *HIF1A* and *HIF2A*. **(E)** GSEA plot of the enrichment pathway on genes at 14q associated with corresponding SCNAs. OXPHOS, Oxidative phosphorylation; EMT, Epithelial-Mesenchymal Transition; Pos, Positive; Neg, Negative; Cor, Correlation coefficient; NES, Normalized Enrichment Score.

The analyses described above have revealed immuno-genomic features associated with ccRCC progression, but differences in the genome and immune microenvironment features between aggressive and metastatic ccRCC were not observed. Towards this end, we investigated differences in biological processes among the ccRCC phenotypes. GSEA showed that genes upregulated in aggressive and metastatic ccRCC were enriched in pathways associated with the immune response (Interferon (IFN)-γ response and allograft rejection) and cell cycle (E2F targets; **Figure 7A**). Notably, only cell cycle pathways differed significantly between aggressive and metastatic ccRCC. Furthermore, the pathways enriched for genes downregulated in aggressive and metastatic ccRCC in the TCGA cohort were associated with oxidative phosphorylation; however, this was not observed in the ICGC cohort.

We analyzed the correlation between the ssGSEA scores of the above biological processes and indicators related to extrinsic and intrinsic immune escape to explore whether these biological processes were associated with features of the immune microenvironment of ccRCC. Due to the very strong correlation between IFN-γ response pathway and allograft rejection pathway (*r* = 0.95; **Figure S4B**), we recalculated the ssGSEA scores after merging their gene sets, named immune infiltration score, and the ssGSEA score of the E2F targets pathway was defined as the cell cycle score. The immune infiltration score well distinguish between localized from non-localized ccRCC and had a significant positive correlation with immune microenvironmental features associated with ccRCC, including infiltration of CD8^+^ TILs and Tregs, the immunostimulatory factors *PDCD1* and *LAG-3*, and the immunostimulatory factor *CD27* (**Figure 7B**; **Figure S4C**). The cell cycle scores distinguished between aggressive and metastatic ccRCC but were not correlated with the above extrinsic immune escape features (**Figure 7B**). Conversely, it had a strong positive correlation with CTA score and HRD score, whereas the immune infiltration score correlated weakly with the immunogenicity indicators (**Figure S4C**). Since ccRCC progression was accompanied by genomic alterations and dynamic changes of the immune microenvironmental features, we explored the association between genomic alterations and immune infiltration in ccRCC. The results showed that all recurrent or low-frequency differential mutations in ccRCC were not associated with the immune infiltration score and immune microenvironmental features related to ccRCC progression. At the SCNA level, a moderate positive correlation was observed between the immune infiltration score and Del(14q) (*r* = 0.33), and the cell cycle score showed no obvious correlation with either differential SCNA (all *P* > 0.05; **Figure 7C**). Overall, the immune infiltration score reflected the dynamic changes in the immune microenvironment characteristics during ccRCC progression, while the cell cycle score reflected the aggressiveness and metastatic ability of ccRCC.

### Genomic Copy Number Alterations as a Driving Factor of Change in the Immune Microenvironment of ccRCC

Integrative multi-omics analysis identified an association between Del(14q) and immune features in ccRCC. Therefore, we further explored the potential mechanisms by which Del(14q) drives changes in the immune microenvironment of ccRCC. The genes mapped to chromosome 14q were selected and ranked according to their correlation with the immune infiltration scores and subsequently subjected to GSEA analysis. The results showed that these genes were only enriched in the VEGFA-VEGFR2 signaling pathway, although it was insignificant (**Figure 7D**). The alterations in the leading-edge genes’ expression levels in this pathway could be explained by Del(14q), except for *HSP90AA1* and *RHOJ* (**Figure 7D**). Besides, *HIF1A* at chromosome 14q and *HIF2A* at chromosome 2p were thought to be upstream regulators of the VEGFA-VEGFR2 signaling pathway (46, 47). Correlation analysis revealed significant correlations between *HIF1A* and *HIF2A* and the leading-edge genes in the VEGFA-VEGFR2 pathway except for *HSP90AA1* and *RNP* (**Figure 7D**), where *HIF1A* expression could be explained by Del(14q) (**Figure S5A**). We divided ccRCC into two groups according to the deletion status of chromosome 14q and performed enrichment analysis for differentially expressed genes between them to further confirm the correlation between Del(14q) and altered ccRCC immune microenvironment. The results showed that most pathways enriched by upregulated genes in the Del(14q) group were associated with the immune response (**Figure S5B**). Furthermore, the genes mapped to *14q* were sorted according to the correlation between their transcription level and the corresponding copy number and subsequently subjected to GSEA analysis. Interestingly, the VEGFA-VEGFR2 signaling pathway was enriched significantly among these genes (**Figure 7E**). Downregulation of their expression caused by Del(14q) could lead to downregulation of the VEGFA-VEGFR2 pathway. Overall, Del(14q) was potentially involved in driving immune cell infiltration and extrinsic immune escape in ccRCC, possibly through the VEGFA-VEGFR2 signaling pathway.

### Cell Cycle Pathways are Associated With Tumor Progression and Response to Immunotherapy in ccRCC

**Figure 8:**
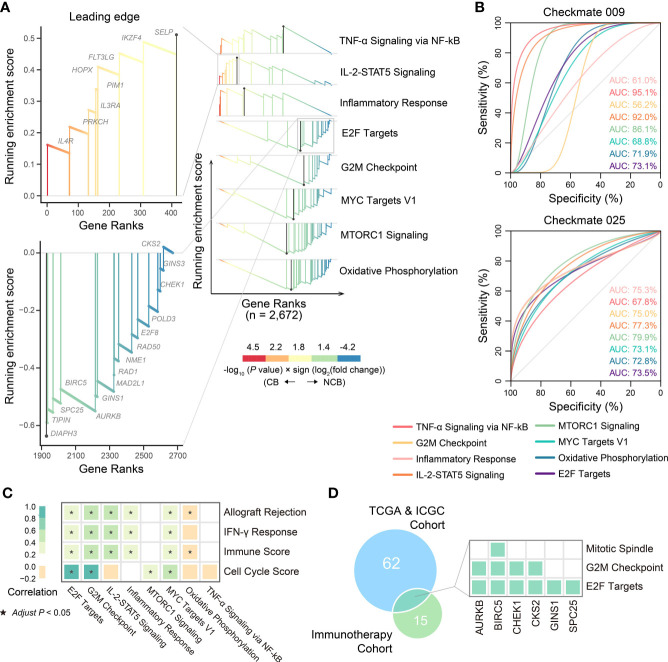
The biological pathways related to immunotherapeutic response. **(A)** The differential biological processes between CB group and NCB group of ccRCC in immunotherapy cohort and Zoom-in plot showing leading edge genes of IL-2-STAT5 signaling and E2F targets pathways. **(B)** ROC curves and corresponding AUC of differential biological processes between CB group and NCB group of ccRCC in Checkmate009 (top) and Checkmate025 (bottom) cohorts. **(C)** Pearson correlation of ssGSEA scores of biological pathways related to ccRCC progression with those related to immunotherapeutic response. **(D)** Overlap of the leading genes of E2F targets related to ccRCC progression and those related to immunotherapeutic response, and the hallmark pathways covered by them.

**Figure 9:**
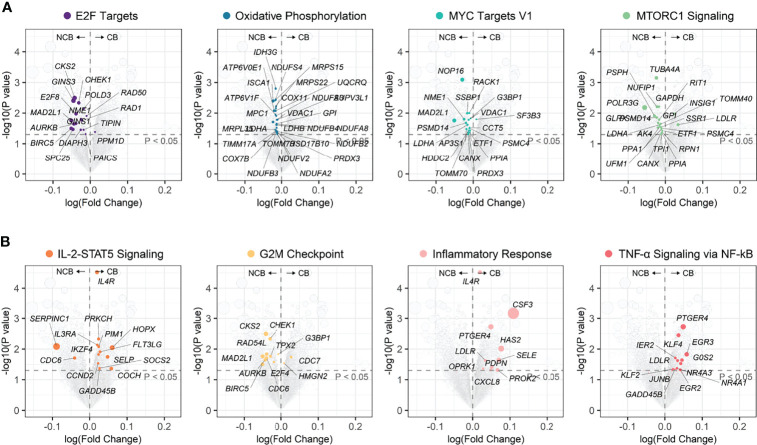
Volcano plots of differential genes between CB group and NCB group of ccRCC in the immunotherapy cohort. **(A)** Position in the volcano plot of genes involved in biological pathways upregulated in the NCB group. **(B)** Position in the volcano plot of genes involved in biological pathways upregulated in the CB group.

We reassessed whether an association existed between ccRCC progression and the immunotherapeutic response, searching for related novel biological pathways or markers. First, differential gene expression analysis was performed between the CB and NCB groups in the immunotherapy cohort. GSEA analysis showed that the upregulated genes in the CB group were enriched in immune-related pathways (e.g., IL-2-STAT5 signaling, TNF-α signaling *via* NF-kB, and inflammatory response), while the downregulated genes were mainly enriched in cell proliferation (e.g., E2F targets, G2M checkpoint, MYC targets V1, and mTORC1 signaling) and oxidative phosphorylation pathways (**Figures 8A**, **9**). Subsequently, we extracted the leading-edge genes in each pathway and analyzed the ability of the ssGSEA scores to predict the immunotherapeutic response efficacy of each pathway. The results showed that the IL-2 STAT5 signaling pathway (AUC = 0.92 and 0.77, respectively) and mTORC1 signaling (AUC = 0.86 and 0.80, respectively) had a strong predictive power in the Checkmate 009 and Checkmate 025 cohorts (**Figure 8B**).

**Figure 10:**
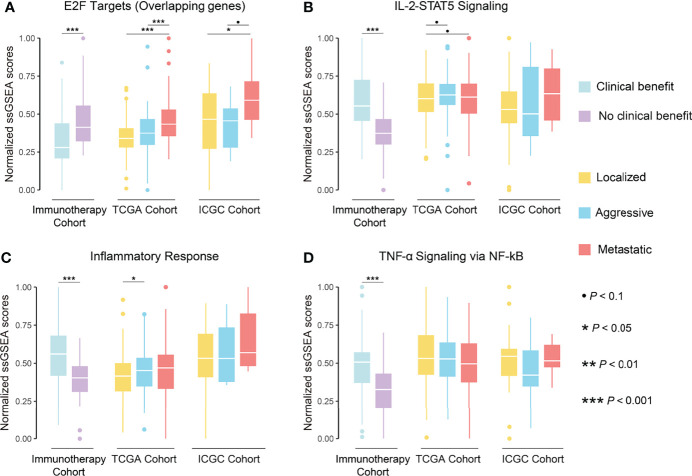
Boxplot showing the differences in normalized ssGSEA scores of overlapping genes of E2F targets pathway **(A)**, IL-2-STAT5 signaling pathway **(B)**, inflammatory response pathway **(C)**, and TNF-α signaling *via* NF-kB pathway **(D)** between phenotypes in TCGA and ICGC cohorts and between groups in immunotherapy cohort.

These results suggested an association between biological pathways related to ccRCC progression and immunotherapeutic response. Further correlation analysis revealed that the cell cycle score was strongly correlated with the immunotherapeutic response-related E2F targets (*r* = 0.91) and G2M checkpoint pathway scores (*r* = 0.97), while the immune infiltration score was moderately correlated with the IL-2-STAT5 signaling pathway score (*r* = 0.46; **Figure 8C**). The strong correlation between the cell cycle score and the E2F targets pathway prompted us to analyze the degree of overlap between their gene sets. Six genes were present in both gene sets and, in the hallmark gene set, they were only present in the pathway related to cell proliferation (**Figure 8D**; **Table S9**). Further, the ssGSEA score consisting of these six genes was significantly higher in metastatic ccRCC than in non-metastatic ccRCC in both cohorts and higher in the NCB group than the CB group in the immunotherapy cohort (**Figure 10A**). Additionally, we found no overlapping genes between the immune infiltration score and either immune-related pathway in the immunotherapy cohort (**Table S9**), which was not associated with ccRCC progression in either cohort (**Figures 10B–D**).

## Discussion

The current stratified treatment strategy for ccRCC is based primarily on the tumor stage. Although non-metastatic ccRCC could undergo radical resection, the recurrence rate of aggressive ccRCC is much higher than that of localized ccRCC. The treatment outcome and prognosis become dramatically poorer once the ccRCC has invaded nearby tissues and metastasized. Although the ICIs had shifted the therapeutic paradigms for metastatic ccRCC, durable benefit was limited to a small number of patients, and traditional biomarkers did not effectively predict response to ICIs in ccRCC. Therefore, the aim of this study was to reveal the immuno-genomic landscape of ccRCC progression and its association with immunotherapeutic response and prognosis, forming the foundation for developing improved early diagnosis, treatment, and prognosis prediction. As expected, we identified genomic alterations that might drive ccRCC progression and accompanying changes in immune microenvironmental features, and found the potential mechanism by which Del(14q) drives changes in the immune microenvironment of ccRCC and biological pathways associated with immunotherapeutic response.

The occurrence and development of tumors are thought to be driven by somatic genome alterations with key roles in many cellular processes, including cell cycle, apoptosis, and DNA damage repair (39, 48, 49). Previous studies found an association between *BAP1* and *SETD2* mutations and metastasis and poor prognosis in ccRCC (50–52). However, we only found an association between *SETD2* mutations and ccRCC progression in both cohorts rather than just metastasis. We speculate that *SETD2* mutations contribute more to the aggressive ability than the distant metastatic ability of ccRCC. Recently, *SETD2* mutations were found to promote ccRCC progression through various mechanisms such as cellular autophagy inhibition, DNA repair inhibition, and genomic stability perturbation (53, 54). Previous studies considered Del(14q) and Del(9p) as ccRCC metastasis drivers, while Del(3p) and Amp(5q) initiated ccRCC development (6, 50, 55). This study found that Del(14q), Del(9p21.3), and Del(9p23) were possible progression drivers in ccRCC rather than just metastasis. Likewise, we found that Del(9q) and Del(4q24) were ccRCC progression drivers. Several studies have shown that *HIF1A* was a cancer suppressor gene at Del(14q) in ccRCC (56, 57). Shen et al. found that Del(14q)-related downregulation of *HIF1A* promoted renal carcinoma growth on mice tumor xenograft (57). Recent research found that *HIF1A* preferentially drove ccRCC initiation rather than progression, and it not necessarily was a target on Del(14q) in ccRCC, echoing our findings (58, 59). However, our results should be interpreted with caution as *HIF1A* transcript levels might be regulated by other factors such as VHL deletion, which diminish the effect of Del(14q) on it (60). We found that *CDKN2A* and *CDKN2B*, traditional tumor suppressors at 9p21.3 (61, 62), struggled to act as tumor suppressors in ccRCC with Del(9p21.3). Indeed, *CDKN2A* overexpression in various tumors had long been noted, and interpreted as a result of dysregulated feedback associated with of the pRb pathway (62). The relationship of *CDKN2A* and *CDKN2B* expression with the corresponding SCNA levels seemed to indicate that complex pathways regulated their transcription levels and that their tumor suppressor properties were not evident in ccRCC. *MTAP* is another tumor suppressor in Del(9p21.3) (63–65). Xu et al. found that MTAP reversed epithelial mesenchymal transition and inhibited migration and invasion of renal cell carcinoma cells (66). Our findings suggested that *MTAP* was a suitable target for Del(9p21.3) in ccRCC. *DAPK1*, *FBP1*, and *TSC1* are well-known targets on Del(9q), a SCNA related to ccRCC progression (67–69). We found that these three genes were good targets for Del(9q) in ccRCC as their transcript levels significantly correlated with SCNA levels. Li et al. found that *PTPRD* deletion on 9p23 was associated with poor prognosis in ccRCC (70). However, *PTPRD* expression was not associated with SCNA levels or tumor progression in our study. *CXXC4* and *TET2* at 4q24 also act as tumor suppressors (41, 42). Interestingly, we found them to play a tumor suppressor role in ccRCC even though their expression was influenced by other factors more than SCNAs. Overall, certain tumor suppressor genes, well-known in other cancers, might not play an obvious role in regulating tumor progression in ccRCC.

The immune microenvironment of ccRCC has received considerable attention as increasing evidence suggested that it plays a crucial role in anti-cancer immunity (71). Previous studies have shown that ccRCC were inflammatory tumors accompanied by high infiltration of exhausted CD8^+^ TILs and immunosuppressive cells (72). We found high infiltration levels of CD8^+^ TILs and Tregs in early ccRCC, and increasing *PDCD1* and *LAG-3* levels with ccRCC progression in both cohorts. Braun et al. found that high expression of *PDCD1* and *LAG-3* on tumor-infiltrating CD8^+^ T cells in advanced ccRCC by using single-cell sequencing, echoing our above finding (73). High CD8^+^ TIL levels were associated with a good prognosis in most tumors (9). However, several studies have found that high infiltration of CD8^+^ TILs and Tregs, and high levels of *PDCD1* expression were associated with a poor prognosis in ccRCC, consistent with our findings (74–76). The combination of high exhaustion of TILs, high infiltration of immunosuppressive cells, and high expression of immunosuppressive molecules in aggressive and metastatic ccRCC suggested that ccRCC progression was accompanied by extrinsic immune escape. Functional enrichment analysis in this study indicated that ccRCC progression was accompanied by upregulation of immune-related pathways, including the IFN-γ response and allograft rejection. Several studies have found that IFN-γ response pathway had dual roles in tumor development, with pro- and anti-carcinogenic activities (77). Hakimi et al. observed that upregulation of IFN-γ response pathway in ccRCC was associated with high CD8^+^ T-cell infiltration and exhaustion (78). The allograft rejection pathway upregulation might be caused by the overlapping genes with ccRCC inflammatory profile and the IFN-γ response pathway (79). The increase in the immunostimulatory factors CD27 and IFNG seems to represent antitumor immunity activation (44). However, whether ccRCC progression is accompanied by antitumor immunity activation needs further investigation. Several studies have indicated that the pathway mediated by CD27 and its ligand CD70 was involved in tumor growth and induction of lymphoid apoptosis (80, 81). CXCR3 promoted T-cell migration to the tumor core (45), and its mediated CXCR3-CXCL10 signaling pathway drove ccRCC metastasis (82). Based on the immune editing theory proposed by Schreiber et al. (43), the unique immunosuppressive microenvironment features of ccRCC were potentially driven by tumor intrinsic factors. Several reviews have suggested that the immunogenicity of tumors was determined by the production of neoepitopes by tumor genome alterations and the presentation of neoantigens (83, 84). Indicators describing genomic alterations and heterogeneity such as TMB, FGA, ITH, CTA, and HRD have been used to measure the immunogenicity of tumors (22, 26–30). However, none of these was related to the immune microenvironmental features of ccRCC. Instead, CTA score and HRD score are related to the upregulation of E2F targets pathway in ccRCC. It should be noted that the possibility that CTA score and HRD score reflect tumor aggressiveness and metastasis rather than immunogenicity in ccRCC was not excluded. Furthermore, we observed no differences in the antigen-presentation processes, which is closely related to tumor immunogenicity, during ccRCC progression, although this finding was only based on the differential expression of antigen-presenting molecules. These findings seem to suggest that ccRCC might evade immune recognition during progression predominantly by extrinsic immune escape mechanism, although we could not exclude bias in the results due to inaccurate assessment of indicators measuring the immunogenicity and antigen presentation of ccRCC.

Several multi-omics studies have suggested that tumor-intrinsic immune regulation could be triggered by certain genomic alterations (24, 85). In this study, only Del(14q) correlated significantly with the immune microenvironment features and immune-related pathways in ccRCC. Del(14q) might drive considerable immune cell infiltration and exogenous immune escape in ccRCC by downregulating the VEGFA-VEGFR2 signaling pathway. Clark et al. found in the CPTAC cohort that the CD8^+^ inflamed phenotype of ccRCC showed extensive infiltration and exhaustion of CD8^+^ TILs, was associated with Del(14q), and was accompanied by downregulation of angiogenesis-related signaling pathways as was also observed in the study of Hakimi et al. (78, 86).

Identification of biological features associated with response to immunotherapy could help predict its efficacy and optimize treatment regimens. Unfortunately, indicators used to predict response to ICIs in other cancers, including *PD-L1* expression, TMB, and the extent of CD8^+^ T cell infiltration, did not work in ccRCC (11–13). This could be because pathways associated with immunotherapeutic response and immune infiltration scores associated with ccRCC progression did not overlap, as shown by our functional enrichment analysis. Interestingly, most relevant to immunotherapeutic response are the upregulation of the IL-2 STAT5 signaling pathway and the downregulation of the mTORC1 signaling pathway, both have long been applied in systemic ccRCC treatment. IL-2 therapy promotes the development and killing activity of effector TILs and has been shown to benefit patients with aggressive and metastatic ccRCC (87). The mTORC1 signaling pathway is frequently activated in ccRCC and involved in the proliferation and metabolism of tumor cells (88). mTOR inhibitors are currently used as a recommended second-line regimen for ccRCC (2, 3). Therefore, upregulation of IL-2 STAT5 signaling pathway and downregulation of mTORC1 signaling pathway in the CB group suggested that mTOR inhibitor or IL-2 therapy might help improve the therapeutic response to ICIs, which deserves further research. We further found that the E2F TARGETS pathway was negatively associated with immunotherapeutic response and positively with ccRCC progression. Notably, both the immune features and, at least, the tumor cell proliferation features influenced the response to immunotherapy in ccRCC. Recently, Pabla et al. reported tumor cell proliferation as a factor influencing the response of non-small cell lung cancer to immunotherapy (89). Combined with the recent preliminary results of neoadjuvant immunotherapy for ccRCC (90), we speculate that similar or even better immunotherapy efficacy is achieved in non-metastatic and metastatic ccRCC due to the low proliferation activity of tumor cells in non-metastatic ccRCC. Furthermore, Li et al. described in their recent study that enhanced cell cycle activity in cancer cells lead to immunotherapy resistance and combination of pharmacological inhibition of the cell cycle might help to further improve the response to immunotherapy (91). However, evidence on the correlation between the proliferation ability of ccRCC and immunotherapy response is lacking, which merits more exploration and validation.

Although this study provided a comprehensive description of ccRCC immuno-genomic features and mined for genomic alterations affecting the immune microenvironment of ccRCC and the biological pathways associated with its immunotherapeutic response, the study had some limitations that await improvement. We classified metastatic ccRCC as tumors with T_4_N_any_M_0_ or T_any_N_any_M_1_ following the AJCC staging. However, ccRCC with distant metastases and locally advanced ccRCC have different tumor evolution patterns even though they are both Stage IV tumors, possibly leading to bias in the results (6). The small number of Stage IV ccRCC cases with distant metastases in both cohorts did not allow for a valid separate analysis, which will be refined by expanding the sample size in future studies. Second, all the ccRCC samples included in this study were sampled from a single location, ignoring the intratumoral heterogeneity of ccRCC. We attempted to reduce the inherent bias from tumor heterogeneity by analyzing large-sample multi cohorts. Finally, all the conclusions in this study were drawn from *in silico* comparative genomics analyses. Experimental and clinical verification is needed to facilitate the clinical translation of the findings.

In conclusion, this study provided important clues to the intrinsic mechanisms driving the progression of ccRCC and associated changes in the immune microenvironment. Moreover, the genomic alterations involved in immune regulation and biological pathways associated with immunotherapeutic responses demonstrated potential clinical value, providing new insights for developing precise therapeutic strategies and efficient prediction protocols for patients with ccRCC.

## Data Availability Statement

The datasets presented in this study can be found in online repositories. The names of the repository/repositories and accession number(s) can be found in the article/**Supplementary Material**.

## Author Contributions

EL, PZ, YL and YY conceived the idea for the research and wrote the manuscript. EL and CY created the figures. All authors read, edited and approved the manuscript.

## Funding

This work was supported by the Science and Technology Project of Guangzhou (grant no. 202002030060), Guangdong Basic and Applied Basic Research Foundation (grant no. 2020A1515011473), and National Natural Science Foundation of China (grant no. 82002528).

## Conflict of Interest

The authors declare that the research was conducted in the absence of any commercial or financial relationships that could be construed as a potential conflict of interest.

## Publisher’s Note

All claims expressed in this article are solely those of the authors and do not necessarily represent those of their affiliated organizations, or those of the publisher, the editors and the reviewers. Any product that may be evaluated in this article, or claim that may be made by its manufacturer, is not guaranteed or endorsed by the publisher.

## Supplementary Material

Supplementary Figure 1 **(A)** Kaplan–Meier curves of OS for stages of ccRCC in ICGC cohort. **(B)** Kaplan–Meier curves of OS for phenotypes of ccRCC in ICGC cohort. **(C)** Time-dependent area under the ROC curve of stages and phenotypes for the OS in ICGC cohort. **(D)** Kaplan–Meier curves of OS for stages of KIRP. **(E)** Kaplan–Meier curves of RFS for stages of KIRP. **(F)** Kaplan–Meier curves of OS for stages of KICH. **(G)** Kaplan–Meier curves of RFS for stages of KICH.

Supplementary Figure 2 **(A)** Waterfall plot with recurrent and differential mutations among phenotypes of ccRCC (right) and forest plot of the univariate Cox regression analysis of mutations for OS (left) in ICGC cohort. **(B)** Forest plot of the univariate Cox regression analysis of mutations and SCNAs for OS and RFS in TCGA cohort.

Supplementary Figure 3 **(A)** Kaplan–Meier curves for OS of ccRCC with high and low levels of immunogenicity indicators. **(B)** Kaplan–Meier curves for RFS of ccRCC with high and low levels of immunogenicity indicators

Supplementary Figure 4 **(A)** Correlation of immunogenomic indicators with indicators related to extrinsic immune escape. **(B)** Correlation of ssGSEA scores of the pathways related to ccRCC progression. **(C)** Correlation of ssGSEA scores of the pathways related to ccRCC progression with indicators related to immune escape.

Supplementary Figure 5 **(A)** Boxplot showing the expression differences of *HIF1A* and *HIF2A* between Del(14q) and wild-type groups in TCGA cohort. **(B)** The differences in biological processes between Del(14q) and wild-type groups in TCGA cohort. NES: Normalized enrichment score. OXPHOS, Oxidative phosphorylation; EMT, Epithelial-Mesenchymal Transition; NES, Normalized Enrichment Score.
