# Supplementary figures and images for: Integrative Analysis of the Genomic and Immune Microenvironment Characteristics Associated With Clear Cell Renal Cell Carcinoma Progression: Implications for Prognosis and Immunotherapy
Source: Front Immunol. 2022 May 23;13:830220. doi: 10.3389/fimmu.2022.830220 (PMC9168804; doi:10.3389/fimmu.2022.830220)

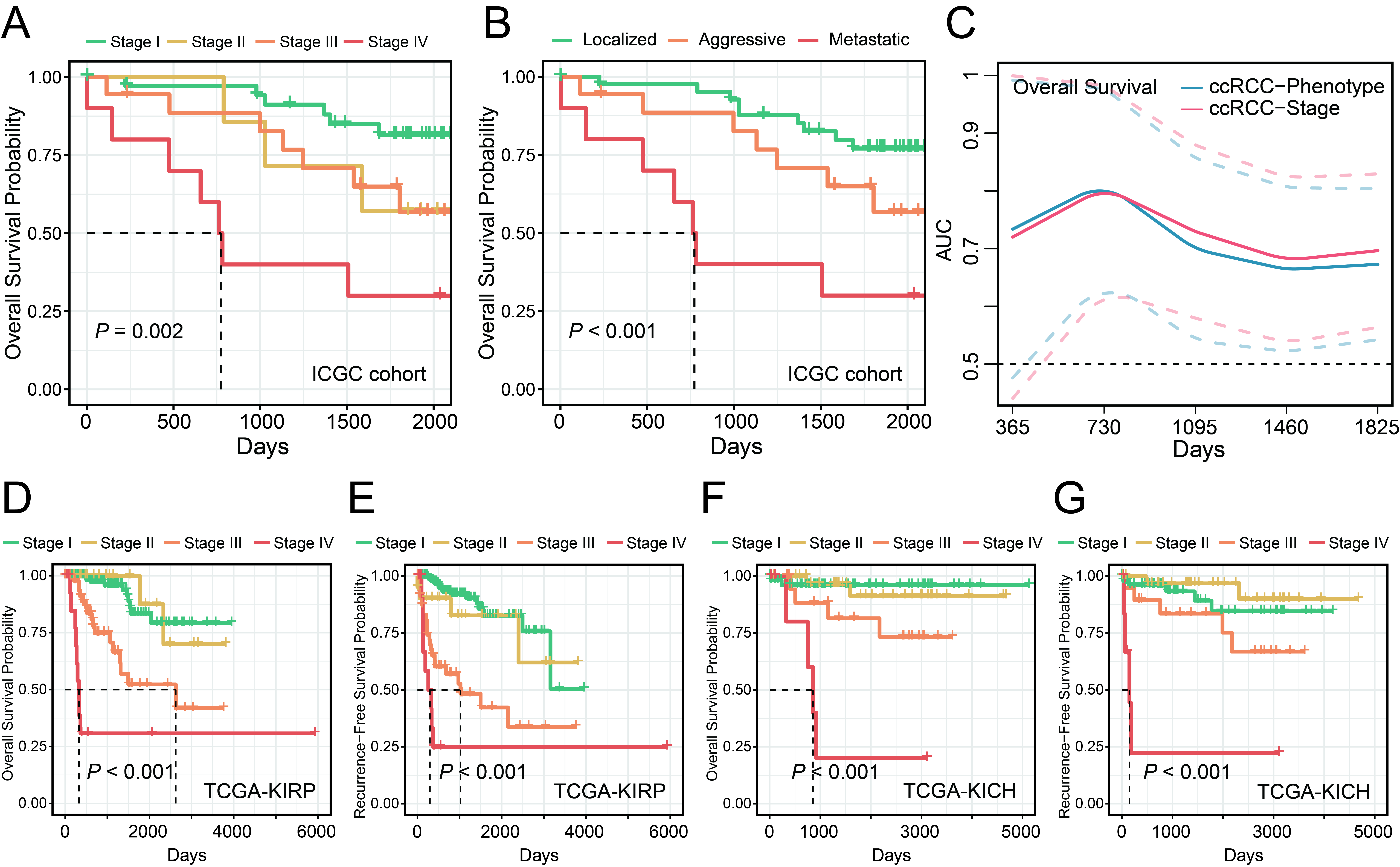

Supplement: Supplementary Figure 1 — (A) Kaplan–Meier curves of OS for stages of ccRCC in ICGC cohort. (B) Kaplan–Meier curves of OS for phenotypes of ccRCC in ICGC cohort. (C) Time-dependent area under the ROC curve of stages and phenotypes for the OS in ICGC cohort. (D) Kaplan–Meier curves of OS for stages of KIRP. (E) Kaplan–Meier curves of RFS for stages of KIRP. (F) Kaplan–Meier curves of OS for stages of KICH. (G) Kaplan–Meier curves of RFS for stages of KICH. [file Image_1.jpeg]

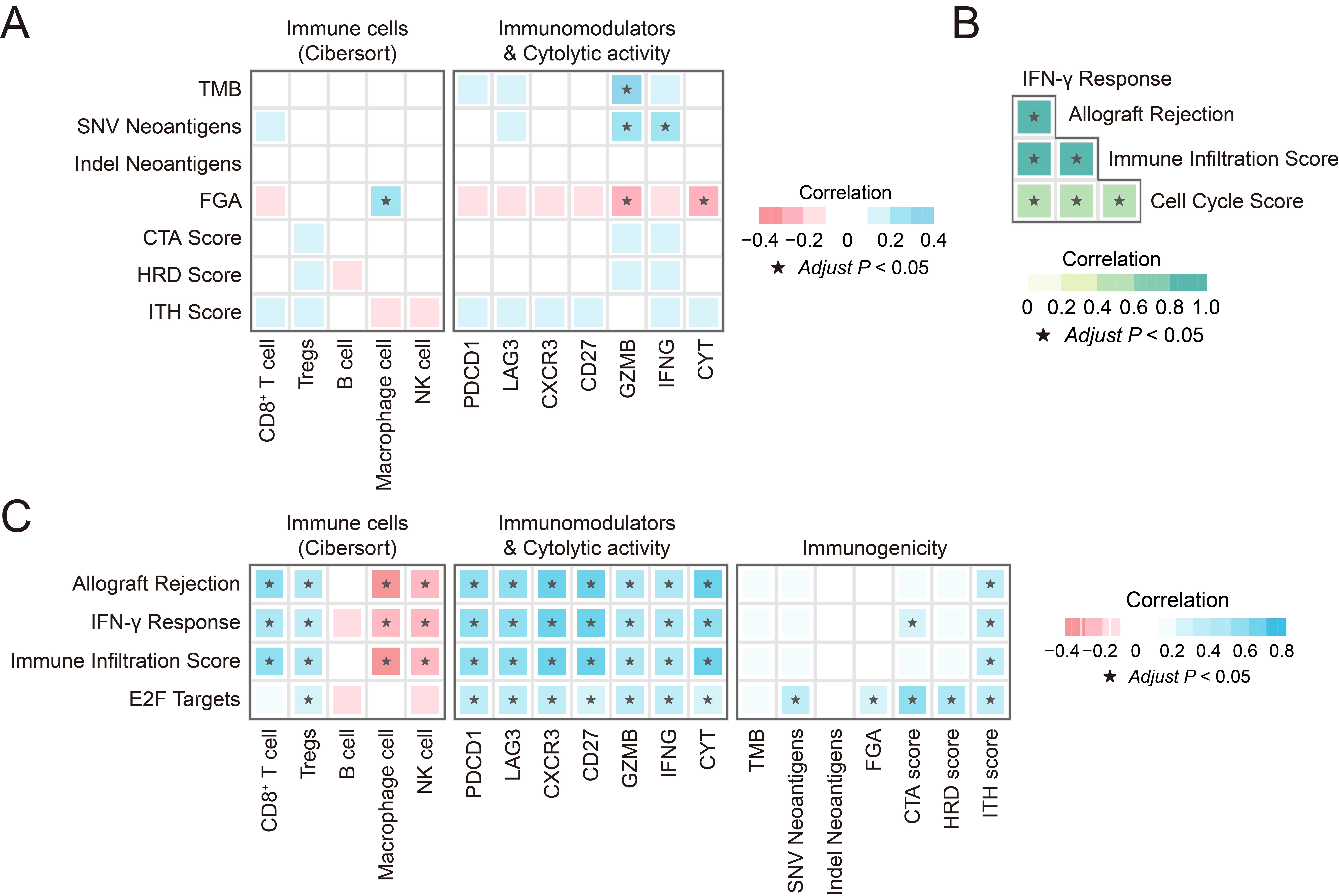

Supplement: Supplementary Figure 4 — (A) Correlation of immunogenomic indicators with indicators related to extrinsic immune escape. (B) Correlation of ssGSEA scores of the pathways related to ccRCC progression. (C) Correlation of ssGSEA scores of the pathways related to ccRCC progression with indicators related to immune escape. [file Image_4.jpg]

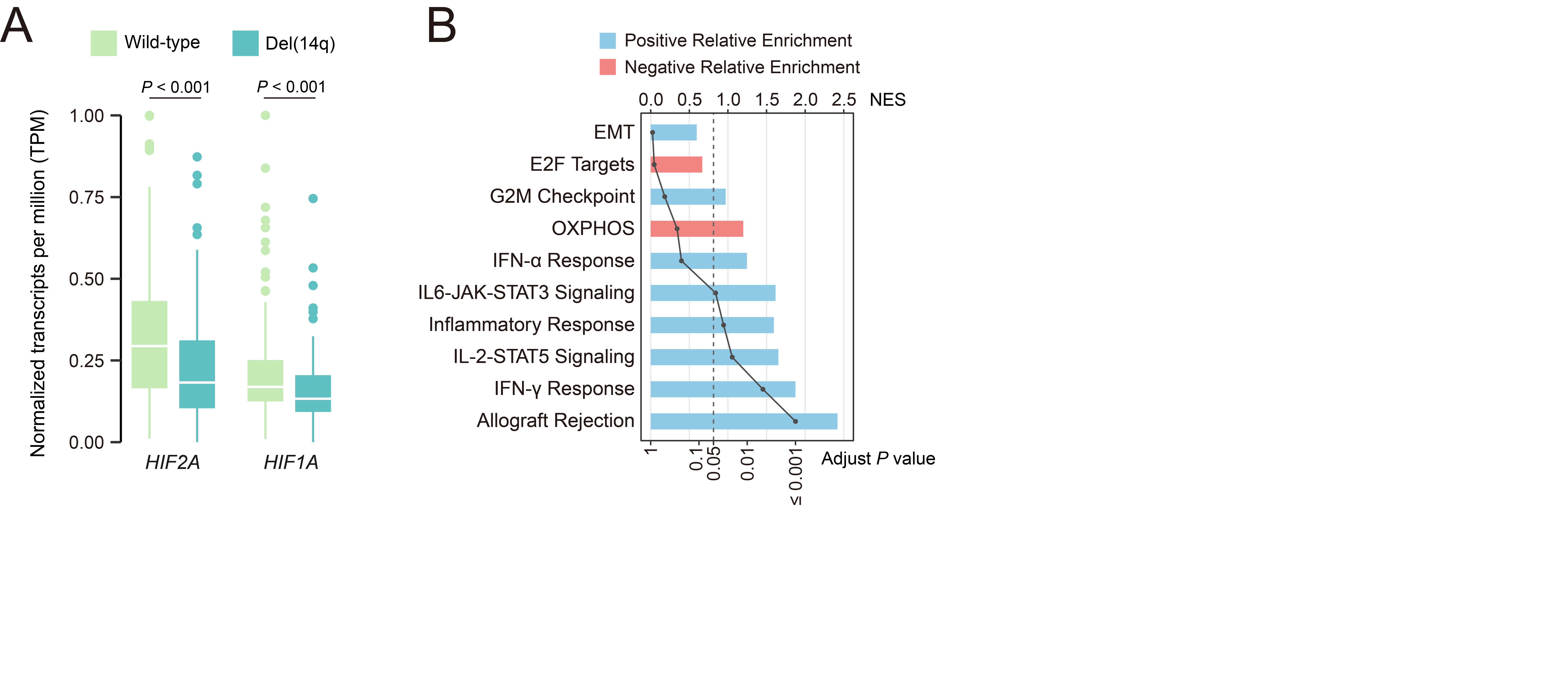

Supplement: Supplementary Figure 5 — (A) Boxplot showing the expression differences of HIF1A and HIF2A between Del(14q) and wild-type groups in TCGA cohort. (B) The differences in biological processes between Del(14q) and wild-type groups in TCGA cohort. NES: Normalized enrichment score. OXPHOS, Oxidative phosphorylation; EMT, Epithelial-Mesenchymal Transition; NES, Normalized Enrichment Score. [file Image_5.jpeg]
